# Short-and Long-Term Effectiveness of a Physical Activity Intervention with Coordinated Action between the Health Care Sector and Local Sports Clubs. A Pragmatic Trial in Austrian Adults

**DOI:** 10.3390/ijerph16132362

**Published:** 2019-07-03

**Authors:** Sylvia Titze, Wolfgang Schebesch-Ruf, Christian Lackinger, Lena Großschädl, Albert Strehn, Thomas E. Dorner, Josef Niebauer

**Affiliations:** 1 Institute of Sport Science, University of Graz, Mozartgasse 14, 8010 Graz, Austria; 2 German University of Health and Sports, Vulkanstraße 1, 10367 Berlin, Germany; 3 Austrian Public Health Association, Alser Straße 4, 1090 Vienna, Austria; 4 Social Insurance Authority for Business, Regional Office Styria, Körblergasse 115, 8010 Graz, Austria; 5 Competence Center Health Promotion, Social Insurance Authority for Business, Osterwiese 2, 7000 Eisenstadt, Austria; 6 Department of Social and Preventive Medicine, Centre for Public Health, Medical University of Vienna, Kinderspitalgasse 15/1, 1090 Vienna, Austria; 7 Institute of Sports Medicine, Prevention and Rehabilitation and Research Institute of Molecular Sports and Rehabilitation Medicine, Paracelsus Medical University Salzburg, Lindhofstrasse 20, 5020 Salzburg, Austria

**Keywords:** inter-sectoral collaboration, standardised sports club programme, health care sector, physical activity, counselling

## Abstract

(1) Background: Collaboration between the health care sector and the sports sector to increase physical activity (PA) behaviour among inactive adults is still rare. The objective of the study was to evaluate the short- and long-term effectiveness of a mixed PA intervention on the PA behaviour in an adult population. (2) Methods: In a quasi-experimental study with two follow-up measurements (four and 12 months), adults were contacted by post before their stay in a health resort. During the health resort stay, the intervention group (IG) received PA counselling and a coupon for 12 standardised free-of-charge sessions in a sports club. The participants in the comparator group (CG) received PA counselling and written material. PA was measured with an accelerometer (GENEActive). Linear mixed-effects models were applied to examine the change in PA behaviour, both within and between groups in moderate- to vigorous-intensity PA over time. (3) Results: We obtained at least one follow-up measurement from 217 participants (IG = 167, CG = 50), who were 50% female, with an average age of 53 (±6) years. PA significantly increased from the baseline to the four-month measurement by 58 min./wk (95% CI 36, 80) and to the 12-month measurement by 24 min./wk (95% CI 2, 46) within the IG. No change in PA occurred in the CG. We also found a short-term between-group (IG vs. CG) difference in change over time, but not a long-term difference. (4) Conclusions: The study confirms that a collaboration between the health care sector and local sports clubs is a feasible method of recruiting people into a standardised PA programme and to increase their PA over the long term.

## 1. Introduction

There is solid scientific evidence regarding the positive health effects of regular physical activity (PA) [1]. However, the results of PA monitoring in Austria [2] showed that only 47% of the Austrian adult population met the minimum recommended amount of at least 150 min. of moderate-intensity aerobic physical activity (MPA) per week. Koelen et al. [3] pointed out the increased need to join forces, both within the health care sector and between the health care and other societal sectors, to successfully increase healthy lifestyles at the population level. In line with this, the WHO Regional Office for Europe [4] suggests that one of the six guiding principles to promote PA is the “integrated, multi-sectoral and partnership-based approach”. One possible strategy for establishing alliances between the health care and sports sectors is the use of “Care Sport Connectors” (CSCs), as introduced in the Netherlands [5]. A CSC has both a broker and a facilitator role. The CSC (e.g., exercise physiologist) provides coordinated actions, such as counselling and establishing PA offers, in order to connect inactive people in primary care with PA activities in the community. 

Health resorts is another potential health care setting for contacting physically inactive people and providing PA counselling. In Austria, a health resort can be attended for one to three weeks by adults with risk factors for chronic, non-communicable diseases who are covered by an Austrian social insurance scheme. An individual with such cover can apply for a residential stay in a health resort twice over a five-year period [6].

Smock and Alemagno [7] identified the lack of time and lack of standard guidelines and operating procedures as barriers to referral. Another barrier for PA counselling in the health care setting is neither the target group nor the health staff being familiar with the available PA activities in the neighbourhood of the client. Furthermore, they rarely know whether the PA activity is suitable for the target group [5]. Thus, a specifically designed standardised PA programme that is provided by a sports club in the client’s neighbourhood could be provided in collaboration after PA counselling in the health resort.

Lackinger et al. piloted the feasibility of recruiting patients from the health care sector into target-group-specific exercise courses that are provided by Austrian sports clubs [8]. In this study, 369 (42%) women and men suffering from type 2 diabetes mellitus who filled in the baseline questionnaire finished the study after 12 months. The average PA increase from baseline to 12 months later was 35 min./wk (from 100 to 135 min./wk). However, a limitation of this study with respect to a pragmatic approach was that the intervention was delivered by the project staff and not by the sports club instructors. 

We are not aware of a study in which coordinated measures from social insurance companies have been undertaken to connect inactive adults during their health resort stay with standardised sports club programmes in their home environment. Already existing structures (health resorts and local sports clubs) profiting from each other by establishing an alliance is the advantage of this approach. However, evaluation is needed to examine the effectiveness of such an intervention on PA behaviour. 

The main objective of the current study was to evaluate the short- and long-term effectiveness of a mixed PA intervention (counselling in a health care setting combined with a standardised sports club programme) on PA behaviour.

It was hypothesised that the IG would reflect an increase in PA behaviour over the short term, and that these improvements would be maintained in the long term. It was further hypothesised that those within the IG who regularly attended the standardised sports club programme—named JACKPOT—would have a greater increase in their PA behaviour than those who opted not to attend the JACKPOT programme. 

## 2. Materials and Methods

The methods that were used in the study are outlined here and have been described in more detail elsewhere [6,9]. All of the subjects gave their informed consent for inclusion before they participated in the study. The study was conducted in accordance with the Declaration of Helsinki, and the Ethics Committee of the University of Graz approved the protocol (GZ. 39/86/63 ex 2014/15).

### 2.1. Design, Recruitment and Participants 

We applied a quasi-experimental design with PA measurements at baseline (BL), follow-up 1 (FU1 = 4 months later) and follow-up 2 (FU2 = 12 months later). The participants were recruited by post from 14 defined regions in Styria, a federal state in the south of Austria. One option would have been to make a cluster randomization by randomly assigning regions to the intervention or the control region. However, working closely together with the organized sports, we were advised to purposefully select the intervention regionswith the rational that for the intervention, we needed regions with the capacity to provide infrastructure and instructors. After the intervention regions were selected, control regions were defined not to close to the intervention regions. According to the postal code, the participants from eight regions were allocated to the intervention group (IG) and participants from the other regions were allocated to the comparator group (CG). The recruitment took place between October 2015 and February 2017.

Adults with risk factors for cardio-metabolic or musculoskeletal system diseases who are also covered by an Austrian social insurance scheme can apply for a residential stay in a health resort twice over a five-year period, with the stipulation that the attending physician has to approve the application. In this study, three out of the five main Austrian health insurance companies integrated the contact with the potential study participants into their administrative routine. The companies identified all the adults from the 14 regions within the age range of 30–65 years who were assigned to attend a residential stay in a health resort during the recruitment period. The companies then sent a permission letter for the residential stay and included an invitation for a seven-day accelerometer-based measurement (=baseline) of PA. The two other health insurance companies were not ready to contact their insured parties within their administrative routine, but they agreed that the health resorts would contact the eligible people. Inclusion criteria for the continuation of the study after the baseline measurement were: (1) an age range between 30 and 65 years; (2) a valid baseline accelerometer measurement (data from at least 4 days/wk and for at least 10 h/day); (3) <300 min./wk of baseline moderate-intensity PA/wk or an equivalent combination of moderate-and vigorous-intensity PA/wk. If someone combined moderate- and vigorous-intensity PA/wk the minutes of vigorous-intensity physical activity were multiplied by 2 in order to classify the person; and, (4) written consent to continue the study with PA measurements at four and 12 months after the first measurement.

Intervention settings were 30 health resorts spread over Austria and 15 sports clubs in the eight intervention regions. 

### 2.2. Intervention in the Health Resorts

The following components of the health resort intervention were standardised: invitation letter to potential study participants for a seven-day PA measurement; a PowerPoint presentation about the project for each health resort; the form of the written feedback about the individual PA behaviour; and, at the end of the health resort stay, a phone call to all potential study participants by a project staff member to further inform them about the study. During the health resort stay, all of the participants (IG and CG) with a valid accelerometer measurement received PA counselling. However, the context, when and by whom the information was delivered was not standardised.

The participants of the IG were provided with a coupon for 12 initial free-of-charge JACKPOT sessions in a sports club in their home region. It was intended that this coupon would support the IG participants to start a supervised standardised programme.

The participants of the CG were provided with a booklet about PA and health [10]. Thus, the CG received PA counselling and a booklet, but no voucher, and no invitation to join the JACKPOT programme.

### 2.3. Intervention in the Sports Clubs

The sports clubs that were included in this intervention were typical local sports clubs. At least one sports club provided the JACKPOT programme twice a week in each of the eight intervention regions. However, the majority (90%) of the participants took part in a JACKPOT session once a week. We developed course material with 12 examples of how a JACKPOT session should be structured [11]. The standardised exercise programme consisted of three components: 40-min. endurance training; 30-min. strength training; and, 20-min. behaviour change counselling. The counselling was based on the core model of “COM-B”, the initials of which stand for “capability”, “opportunity”, “motivation”, and “behaviour” [12], indicating that these capacities should be strengthened in order to support the positive PA behaviour change. The maximum number of people in a training group was restricted to 12 people.

During the last free-of-charge JACKPOT session, the participants were encouraged by the sports club instructor to continue the supervised exercise programme. However, for the continuation of the JACKPOT programme, the participants were required to pay a membership fee of €80 per semester. All the sports club instructors were local and attended a two-day in-service training course.

### 2.4. Measures

The primary outcome was the amount of PA per week. Device-based moderate- to vigorous-intensity physical activity (MVPA) was assessed while using a GENEActive triaxial accelerometer (Activinsights, Kimbolton, UK). Participants were asked to wear the device on the non-dominant wrist for seven days. Continuous raw data were recorded at 60 Hz and compiled to 60 s epochs. The criteria for a valid day were ≥10 h of data and for a valid week a minimum wear time of four days a week. Non-wear-time was defined as ≥60 consecutive minutes of zero values. The intensity classification was based on Eslinger et al. [13]: light intensity = 217 to 644, moderate intensity = 645 to 1810, and vigorous intensity ≥ 1811 SVMgs. The minutes of at least 10-min. bouts were summed for the calculation of the time spent in MVPA. The attendance level for the JACKPOT programme is expressed as the number of attended sessions over 12 months. The documentation was completed by the exercise instructor, who filled in an attendance record for every session.

The socio-demographic variables were collected with a self-administered questionnaire.

### 2.5. Statistical Analysis

Comparisons of baseline values between groups were made while using t-tests for continuous variables and chi-squared tests for categorical variables.

The accelerometer data were checked for outliers. In the case of an extreme and probable outlier, we “winsorised” the outlier by replacing it with the next highest score that was not an outlier, plus one. The rational for winsorising is to reduce bias, because the outliers are unrepresentative of the sample as a whole [14].

The effect of the intervention on the primary outcome variable was assessed while using linear mixed-effects models. The essential feature of a random-effects model for longitudinal data is that there is natural heterogeneity across individuals in their response over time, and this heterogeneity can be represented by an appropriate probability distribution [15]. A further advantage of the mixed-effects regression modelling approach is that it does not require each participant to have the same number of measurements, provided that the missing data are random [16] (i.e., there are no systematic differences between participants with complete data when compared with those with missing data after taking the baseline data into account). 

We also stratified the IG into those who attended the sports club programme at least once (IG ≥ 1 JACKPOT) and those who did not participate (IG no JACKPOT), as roughly half of the participants of the IG never attended a JACKPOT session (n = 82) and half attended at least once during the 12 months (n = 85).

The models included random participant-level effects. The variable group (intervention, comparator), time point, and the interaction term group*time were included as fixed effects. Finally, the models were adjusted for sex, age, and BMI, because the maximum likelihood was statistically lower than the crude model. The adjusted models are presented, as the results were similar in the unadjusted and adjusted models.

The intervention effects are reported in terms of mean changes in PA over time within groups (IG, IG ≥ 1 JACKPOT, IG no JACKPOT, and CG) and differences between study groups in the within-group changes over time (IG vs. CG). 

We also ran sensitivity analyses, firstly for participants with valid measurements at BL, FU1, and FU2, and then for people who attended at least six or more JACKPOT sessions over the 12 months. 

For all the analyses, we conducted two-sided tests with the significance level set at *p* < 0.05. Analyses were performed while using IBM SPSS Statistics 25. 

## 3. Results

### 3.1. Recruitment and Study Participants

**Figure 1:**
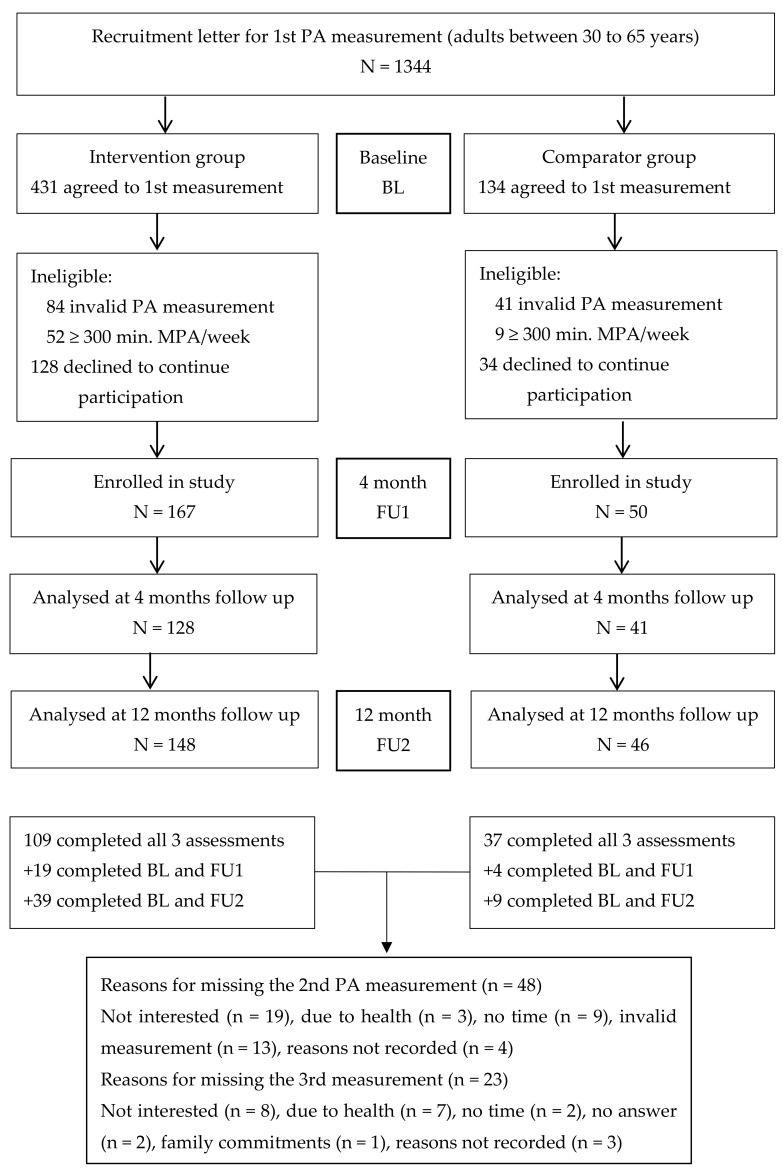
Recruitment and participation flow in the study (BL = baseline, FU = follow-up, N = number).

Figure 1 shows the participant flow through the study. A total of 565 contacted people agreed to the first PA measurement. Of those, 28.7% (162/565) declined to take part in the second measurement. The final sample consisted of 217 participants due to invalid PA measurements and the requirement for doing less than 300 min./wk MPA or an equivalent combination of moderate- and vigorous-intensity PA/wk at baseline. Overall, the study retention was 78% at the four-month PA measurement and 89% at the 12-month PA measurement (some people missed the second but agreed to participate in the third PA measurement). The comparison between the study participants (n = 217) and those with invalid PA measurements and the 162 decliners, respectively (n = 287) revealed no difference in terms of sex, age, education, BMI, and self-perceived fitness. However, the study participants had a higher PA level (104.6 ± 91.1 vs. 79.6 ± 83.3 min. MVPA/wk, *p* = 0.006) than the non-participants.

In terms of the representativeness, study participants were approximately of the same average age as all health resort attendees during the year 2015 in Austria (52.5 years vs. 53.2 years) and the proportion of women was almost equal (51% vs. 53%).

Table 1 lists the characteristics of the study participants. There were no significant differences in the demographic variables or PA behaviour between the IG (as a whole and divided into IG ≥ 1 JACKPOT and IG no JACKPOT) and the CG, and there were no significant differences between the two IG groups. Furthermore, those who participated in both follow-up PA measurements (FU1 and FU2) were not different in their characteristics to those with only one follow-up measurement (FU1 or FU2).

Table 2 lists the participation in the JACKPOT programme across different adherence levels. Almost 60% of those who attended JACKPOT at least once attended all 12 free-of-charge JACKPOT sessions. One-fifth (n = 17) of the IG ≥1 JACKPOT group continued the JACKPOT programme for another half a year (data not shown in Table 2).

### 3.2. Effect of the Intervention on Primary Outcome

There was a statistically significant intervention effect in favour of the IG at the four-month measurement, based on the within- and between-group comparisons. The IG participants increased their PA behaviour by almost an hour per week. The between-group difference (as compared to CG) was 44 min./wk favouring the IG. 

At the 12-month measurement, the IG participants still had a statistically significant increased PA level (close to half an hour per week) when compared to BL. However, the between-group (IG vs. CG) difference in change over time was no longer statistically significant (Table 3).

The results of the sensitivity analyses (firstly, for people with three valid measurements, and then for people who attended at least half of the 12 JACKPOT sessions) were similar to the results of the main analysis.

**Figure 2:**
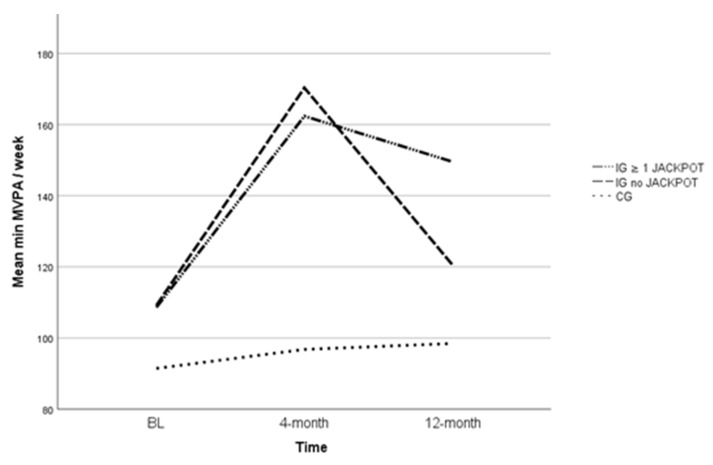
Accelerometer-assessed physical activity (PA) behaviour over time.

The changes in PA behaviour developed differently within the IG ≥ 1 JACKPOT group when compared to the IG no JACKPOT group (Figure 2).

The main difference is that the IG ≥1 JACKPOT group maintained an increased PA level between FU1 and FU2 (no significant decrease in PA), which was not the case for the IG no JACKPOT group. Furthermore, between the BL and the 12-month measurement, there was a borderline significant PA increase in the IG ≥ 1 JACKPOT group. The participants did, on average, about 30 min. more MVPA/wk (*p* = 0.056) when compared to BL. However, the between-group differences were no longer statistically different at the 12-month measurement.

In contrast, those who did not participate in the JACKPOT programme showed significantly decreased minutes of MVPA/wk (within-and between-group comparisons) between FU1 and FU2.

## 4. Discussion

In this paper, we have shown that it is feasible to recruit and retain inactive adults from a health resort setting into a standardised sports club programme provided free of charge. The participants of the CG did not significantly change their PA behaviour over time, whereas the participants of the IG had a significant short- and long-term increase of PA. However, after 12 months, there was no significant difference based on the between-group comparison (IG vs. CG) in change over time. Study retention at four months and 12 months (78% and 89%) can be rated as high, particularly when taking into account that this was not a randomised controlled trial, but was instead a pragmatic trial. The rate is comparable to that of other studies in which (part of) the intervention took place in sports clubs, with the main target being to increase PA and/or weight loss [16,17,18].

Of those who attended the JACKPOT programme, 75% attended ≥ half of the 12 free-of-charge sessions and around 60% completed all 12 sessions. The method of measuring attendance often differs between studies. For example, attendance can be considered as “the proportion of participants completing the exercise programme” or “the proportion of available sessions attended” or “attendance expressed as a proportion of participants reaching certain cut offs” [19]. Williams et al. [20] reported that 12% to 42% of those who attended the first exercise session completed a full course in their systematic review of the effectiveness of exercise-referral schemes. In the German study by Wagner et al. [18], with a standardised “health-directed exercise programme” in a sports club 73% attended at least 67% of the courses over one year. Thus, our attendance rate is within the range of other studies.

After the 12 free-of-charge sessions, 17% of the study participants who attended JACKPOT at least once continued with the JACKPOT programme during the following half a year for a payment of €80. In general, the total adherence was much less than reported for the German sports club study, with an adherence of 76% after three years [18]. The major differences between these two studies are that the intervention in Germany took place in only one sports club, and that the participants did not have to pay any fees during the three-year period. Nevertheless, the JACKPOT programme served well in the adoption and maintenance of regular PA for one-quarter of the initial participants. Furthermore, no injuries were reported during the two years of the JACKPOT programme.

It was surprising that no change in PA behaviour occurred within the CG, although these people received PA counselling during their health resort stay. The PA counselling can be best described as “brief advice”, despite the fact that the participants stayed for one to three weeks at the health resort. Brief advice is defined as verbal advice, discussion, negotiation, or encouragement, with or without written or other support or follow-up. It can vary from basic advice to a more extended, individually focused discussion [21]. In their systematic review, Lamming et al. [22] stated that brief advice can increase self-reported PA, but there is insufficient evidence regarding the long-term impact, or the impact on device-measured PA. In our study, the brief advice, combined with a brochure about PA, did not cause any short- or long-term PA changes. We consider one possible reason for the lack of effectiveness as being worthy of note. It could be that the counsellors felt less motivated with the brief advice, because they could not provide an additional incentive (i.e., a coupon for 12 free-of-charge sessions) to the participant of the CG to encourage the adoption of PA.

In addition, it was unexpected that those who did not participate in a JACKPOT session would equally increase their PA behaviour when compared to the JACKPOT attendees at the four-month measurement. The only difference between the CG and the IG no JACKPOT groups is that the latter group received a JACKPOT voucher. Therefore, the voucher must have been some kind of incentive for the IG participants. Further analysis is necessary to understand why the short-term behaviour change in the IG no JACKPOT group occurred.

The long-term effect of the intervention within the IG ≥ 1 JACKPOT group (borderline significant increase on average of 30 min. MVPA/wk) can be rated as a success. However, the short- and long-term between-group comparison of changes was not significant. This is in line with the outcomes of exercise-referral schemes in primary care. Pavey et al. [23] reported that there was weak evidence of a short-term increase in PA when compared with usual care in their systematic review.

Overall, this study confirms the results from another Austrian study [8] that a collaboration between health care institutions and sports clubs can work to promote health-enhancing PA, and that the participation in a standardised sports club programme increases the PA behaviour within the IG. Leenaars et al. reported similar collaborations [24] in their systematic review, but they were not able to relate the PA outcomes to the different forms of collaboration.

Furthermore, a major advantage of the joined forces between the social insurance scheme and the organized sports was that no new structures had to be established. The recruitment of adults with risk factors was part of a routine treatment/counselling in the health resorts. The local sports clubs are spread over Austria, providing an easy access to the programme with respect to the distance.

The representativeness of the study sample in terms of age and sex, with regard to other health resort clients is one of the strengths of this study. The settings approach to promote PA among inactive adults and the use of already existing settings (health care resorts and sports clubs) to run this pragmatic trial is another strength, from a public health point of view. Furthermore, the PA behaviour was measured with an accelerometer, and short- as well as long-term effects were compared both within and between groups. Finally, the intervention in the sports clubs was standardised.

In contrast, and therefore a limitation of this study, is that the intervention in the health resorts was not standardised. The intervention consisted of two features: feedback for the device-based PA measurement and PA counselling, but how long, by whom, and when the counselling was delivered was not under our control. Those who dropped out of the study had a lower PA level than the study participants. This difference might have lowered the intervention effect, because the likelihood of increasing PA is higher among inactive than active adults [18].

## 5. Conclusions

The widespread network of sports clubs, the recruitment of the study participants by a routine letter from their health insurance companies, and the settings approach in general were preconditions of the study. The study confirms that the coordinated actions were feasible for recruiting people into a standardised PA programme and to increase their PA over the long term. Further research is needed to understand why half of the IG participants never used the coupon for 12 initial free-of-charge sessions.

## Figures and Tables

**Table 1:** Characteristics of participants with at least two accelerometer measurements (baseline (BL) and four or 12 months later).

	Total (n = 217)	IG ≥ 1 JACKPOT (n = 85)	IG no JACKPOT (n = 82)	CG (n = 50)
Sex; N (%)				
Women	111 (51)	47 (55)	36 (44)	28 (56)
Men	106 (49)	38 (45)	46 (56)	22 (44)
Age in years; mean (SD)	53 (6)	53 (7)	52 (6)	53 (7)
Education; N (%)				
≤Obligatory school	22 (10)	11 (13)	7 (9)	4 (8)
Completed apprenticeship	91 (42)	29 (34)	38 (46)	24 (48)
Completed secondary school	79 (36)	30 (35)	31 (38)	16 (32)
Completed college/university	25 (12)	14 (18)	6 (7)	6 (12)
BMI; mean (SD)	26 (5)	27 (4)	27 (5)	27 (4)
BL_MVPA min./wk; mean (SD)	99 (87)	108 (84)	95 (91)	91 (84)

Data are: number (%) or mean (SD); IG ≥ 1 JACKPOT = participants who attended at least one session of the JACKPOT programme; IG no JACKPOT = participants who did not participate in the JACKPOT programme; CG = comparator group; BL_MVPA = baseline moderate- to vigorous-intensity physical activity in minutes; wk = week.

**Table 2:** Attendance for the JACKPOT programme.

Adherence	IG ≥ 1 JACKPOT (n = 85)
	N (%)
≥5 JACKPOT sessions	71 (84)
≥6 JACKPOT sessions	64 (75)
≥12 JACKPOT sessions	49 (58)

**Table 3:** Short and long-term intervention effect on accelerometer-assessed physical activity behaviour.

	Minutes of MVPA/wk by Group			Within-Group Changes			Between-Group Differences vs. Comparator Group		
Groups	Baseline	FU1	FU2	BL−FU1 ^1^	FU1−FU2 ^2^	BL−FU2 ^3^	BL−FU1	FU1−FU2	BL−FU2
	M (SD)	M (SD)	M (SD)	M (95% CI)	M (95% CI)	M (95% CI)			
IG-total	101 (87)	162 (141)	127 (143)	**58 (36 to 80)**	−33 (−60 to 6)	**24 (2 to 46)**	**44 (1 to 88)**	−35 (−86 to 17)	9 (−34 to 52)
IG ≥ 1 JACKPOT	108 (84)	162 (143)	142 (165)	**54 (23 to 86)**	−19 (−59 to 21)	34 (−1 to 68)	41 (−6 to 88)	−21 (−79 to 37)	19 (−31 to 70)
IG no JACKPOT	95 (91)	162 (141)	110 (115)	**61 (29 to 93)**	**−49 (−83 to −14)**	14 (−13 to 40)	**47 (3 to 91)**	**−52 (−100 to −4)**	−1 (−42 to 40)
CG	91 (84)	105 (92)	106 (104)	14 (−12 to 40)	3 (−27 to 34)	15 (−15 to 44)			

From linear mixed models adjusted for baseline sex, age and BMI; MVPA = minutes of moderate- to vigorous-intensity physical activity; IG = intervention group; IG ≥ 1 JACKPOT = study participants who attended at least one session of the JACKPOT programme; IG no JACKPOT = study participants who did not participate in the JACKPOT programme; CG = comparator group; FU1 = follow-up 1; FU2 = follow-up 2; ^1^ mean change at 4-month measurement (reference: baseline); ^2^ mean change at 12 months (reference: 4-month measurement); ^3^ mean change at 12 months (reference: baseline); results in bold indicate a statistically significant difference.
